# Double heterozygous pathogenic variants prevalence in a cohort of patients with hereditary breast cancer

**DOI:** 10.3389/fonc.2022.873395

**Published:** 2022-08-08

**Authors:** Thais Baccili Cury Megid, Mateus C. Barros-Filho, Janina Pontes Pisani, Maria Isabel Achatz

**Affiliations:** Centro de Oncologia Molecular, Hospital Sírio-Libanês, São Paulo, Brazil

**Keywords:** breast cancer, double heterozygous variants, germline panels, hereditary breast cancer, NGS

## Abstract

Hereditary breast cancer (BC) corresponds to 5% of all BC and a larger parcel of early-onset disease. The incorporation of next-generation sequencing (NGS) techniques reduced the cost of molecular testing and allowed the inclusion of additional cancer predisposition genes in panels that are more comprehensive. This enabled the identification of germline pathogenic variants in carriers and the introduction of risk-reducing strategies. It also resulted in the identification of the co-occurrence of more than one germline pathogenic variant in BC genes in some families. This is a rare event, and there are few reports on its impact on cancer risk. We conducted a single-institution retrospective study in which 1,156 women with early onset BC and/or a family history of cancer were tested by a germline multi-gene hereditary cancer panel. Germline pathogenic variants in high- and/or moderate-penetrance BC genes were identified in 19.5% of the individuals (n = 226). The most frequent variants were found in *TP53* (69 of 226; 55 of them represented by p.R337H), *BRCA1* (47 of 226), and *BRCA2* (41 of 226). Double heterozygous (DH) variants were detected in 14 cases, representing 1.2% of all individuals assessed. There were no significant differences in age of BC onset and risk for bilateral BC in DH carriers when compared with those with one germline variant.

## Introduction

Breast cancer (BC) is the most commonly diagnosed cancer in women, with an estimated 2.3 million new cases (11.7%) in Global Cancer Statistics in 2020 (1). A proportion of 5% of BC cases are due to germline pathogenic (P) or likely pathogenic (LP) variants in cancer predisposition genes, and significant progress has been made in the identification of inherited genetic factors underlying hereditary cancers (2). *BRCA1* and *BRCA2* are the most commonly mutated BC susceptibility genes that convey a high-risk of breast and ovarian cancer and represent 25%–28% of hereditary BC (3). In addition, other germline pathogenic variants in genes that confer moderate to high-risk in carriers may also explain other mechanisms of hereditary BC (4).

In Brazil, there is a high prevalence of Li-Fraumeni syndrome due to a founder mutation in TP53 gene, c.1010; p.(Arg337His), that is present in 0.3% of all South and Southeastern Brazilian population (5–7). Age at the time of tumor diagnosis and family history are the best clinical predictors for the identification of carriers (8). Studies have demonstrated that the inclusion of morphologic and immunohistopathologic BC characteristics in models of risk estimation also improves the accuracy in predicting the *BRCA1/BRCA2* status (9).

The non-*BRCA1–* and *BRCA2-*associated hereditary BCs are a heterogeneous group of tumors, with different phenotypes yet to be established. Pathogenic variants in *TP53*, *PTEN*, *CDH1*, *STK11*, and *PALB2* have also been shown to confer high-risk for BC. *ATM*, *CHEK2*, and *BRIP1*, among others, are known to confer moderate risk (10). The clinical impact on BC susceptibility in *NBN*, *PSM2*, and other genes has been described in certain populations, but its impact is still unclear (11–13). The majority of high- and moderate-penetrance genes have an autosomal dominant pattern, in which single heterozygous pathogenic variants lead to cancer risk.

Because the completion of the Human Genome Project, advances in the genomic technology became largely available and the cost of next-generation sequencing (NGS) tests have decreased significantly. In the current context of molecular testing for hereditary cancer predisposition genes, frequently testing only one gene is not enough to detect what is the underlying germline pathogenic variant in a high-risk patient. Therefore, NGS multilane panels became the choice as a less expensive technique, especially if more genes need to be evaluated.

The use of larger NGS panels and the increased use of exome/genome sequencing presented a new challenge to geneticists; the detection of individuals who harbor two or more pathogenic variants in cancer predisposing genes or MINAS (multilocus inherited neoplasia allele syndrome) (14). There are few reports in the literature on the co-occurrence and the cancer phenotypes associated with double heterozygous (DH) pathogenetic variants in cancer predisposing genes and if they combinations appear to have synergistic effects in cancer risk. The co-occurrence of more than one germline pathogenic variant in BC genes is a very rare condition, and its effects on cancer risk in carriers are still unknown.

## Materials and methods

### Study population

This is a single-institution study, comprising patients and family members from the Hereditary Cancer Syndrome Registry at the Oncogenetics Unit, Hospital Sírio-Libanês (HSL), São Paulo, Brazil. Patients were referred to genetic counseling and risk assessment due to early onset BC (before age 40) and/or family history of cancer. All multiple primary cancers were confirmed with histopathological reports or medical records provided by the patients. Multiple primary BCs were considered whenever bilateral disease was present or unilateral disease, which occurred >15 years after the first primary BC.

Certified medical geneticists ascertained all cases and patients received genetic counseling. All participants selected to participate in this study signed an informed consent. All cases were de-identified before data analysis and are non-identifiable. The study was approved by the local Research Ethics Committee (ID1747) and the National Research Ethics Committee (number 39512620.9.0000.5461).

All individuals who performed an NGS germline multi-gene cancer panel (n = 3,030) between January 2013 and December 2021 were ascertained. We selected women with personal history of BC (n = 1156) who had histopathological confirmation. Clinical information, including age at cancer diagnosis, presence of other primary cancer, and BC subtypes (immunohistochemistry for hormone receptors, HER2, and fluorescent *in situ* hybridization for *HER2*), was retrieved from the medical records.

### Genomic variant detection

NGS was carried out by commercial molecular diagnostic laboratories that comprised at least a panel of 12 cancer predisposition BC genes. For the purpose of this study, we included the P and LP variants found on seven high-penetrance genes (*BRCA1*, *BRCA2*, *TP53*, *CDH1*, *PALB2*, *PTEN*, and *STK11*) and four moderate-penetrance genes (*ATM*, *BRIP1*, *CHEK2*, *NBN*, and *PMS2*). Bioinformatic processing was performed at the same certified commercial molecular diagnostic laboratory where NGS was performed. Results were confirmed by a medical geneticist according to recommendations from the American College of Medical Genetics and Genomics (ACMG) and Association for Molecular Pathology (AMP) as well as in public databases, including ClinVar, Clingen, and TP53 IARC database (15, 16).

### Statistical analysis

Statistical analysis was carried out with R (v. 3.4.4; R Project for Statistical Computing). Genomic variants were associated with BC diagnostic age (≤40 or >40 years) and with the development of multiple primary tumors (yes or no) using the Fisher exact test. Age of cancer diagnosis was compared among individuals with no variant detection and with one variant and two or more variants detected using non-parametric Kruskal–Wallis test (Dunn’s *post hoc* test). A two-tailed P < 0.05 value was adopted as significant. Family pedigree illustrations were performed with Progeny software (Delray Beach, FL) (17).

## Results

### Identification of germline variants in BC-risk genes

**Figure 1:**
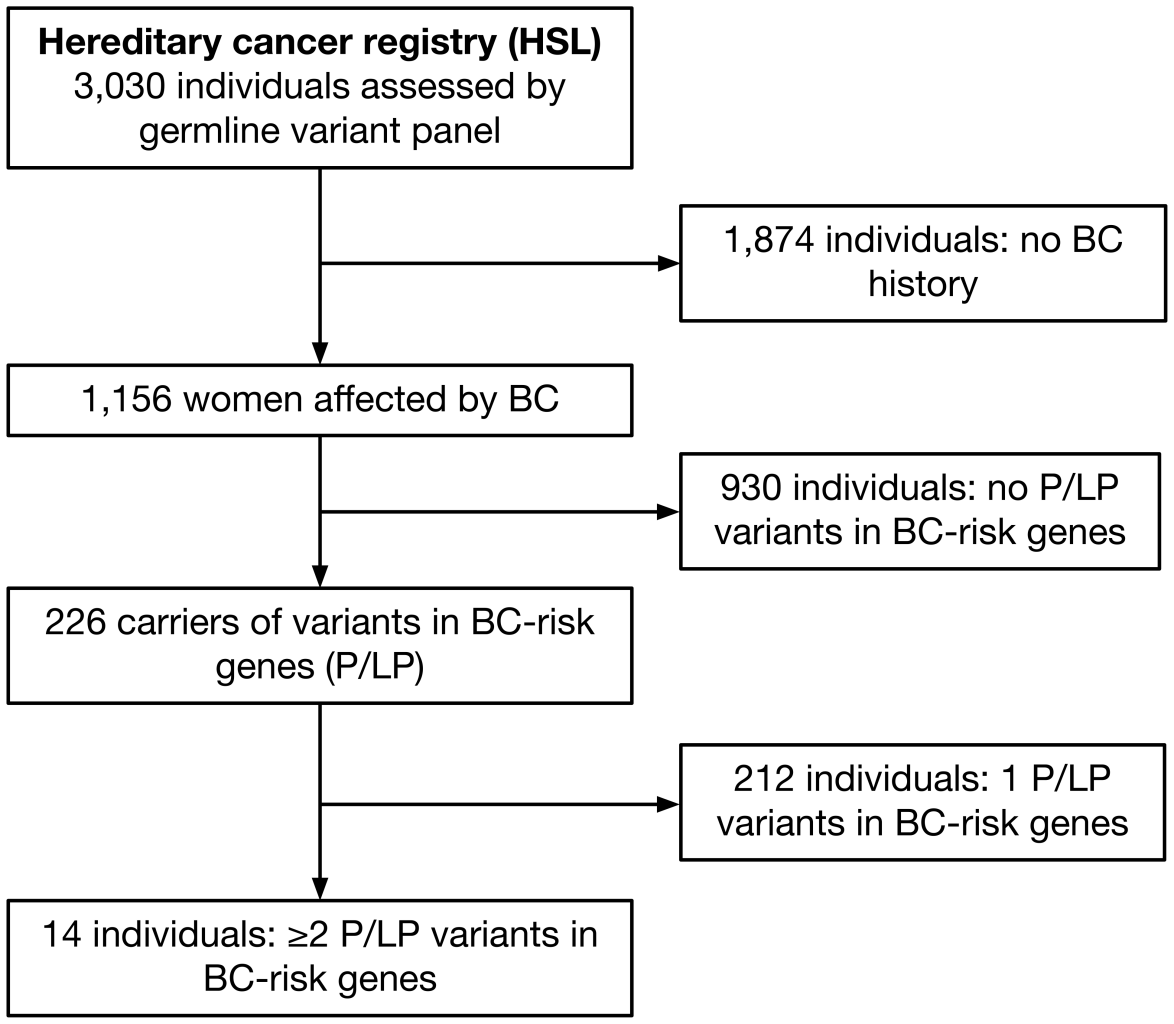
Flowchart summarizing the cohort included in the study. From 3,030 individuals screened from the Hereditary Cancer Registry-Hospital Sírio-Libanês for germline variants by NGS panels, 1,156 had BC and 226 were positive for a pathogenic/likely pathogenic variant in BC-risk genes, 14 of them presented two or more variants.

A total of 1,156 women affected by BC who had undergone a germline BC gene panel testing were included in this study. Germline P or LP variants in moderate and high-penetrance BC genes were identified in 19.5% of all cases (n = 226). A total of 212 individuals presented one P or LP variant and 14 had two or more variants in a BC-risk gene (**Figure 1**).

Germline P and LP variants in *TP53* gene were present in 30.5% of all individuals (69 of 226), 20.8% in *BRCA1* (47 of 226), and 18.1% in *BRCA2* gene (41 of 226). Other variants were identified in *ATM* (14.6% n = 33), *PALB2* (8.0% n = 18), *CHEK2* (5.8% n = 13), *RAD51C* (2.7% n = 6), *PMS2* (2.2% n = 5), *CDH1* (1.8% n = 4), *BRIP1* (1.3% n = 3), *NBN* (0.9% n = 2), and *PTEN* (0.4% n = 1).

### Germline variants and age at BC onset and occurrence of multiple tumors

**Figure 2:**
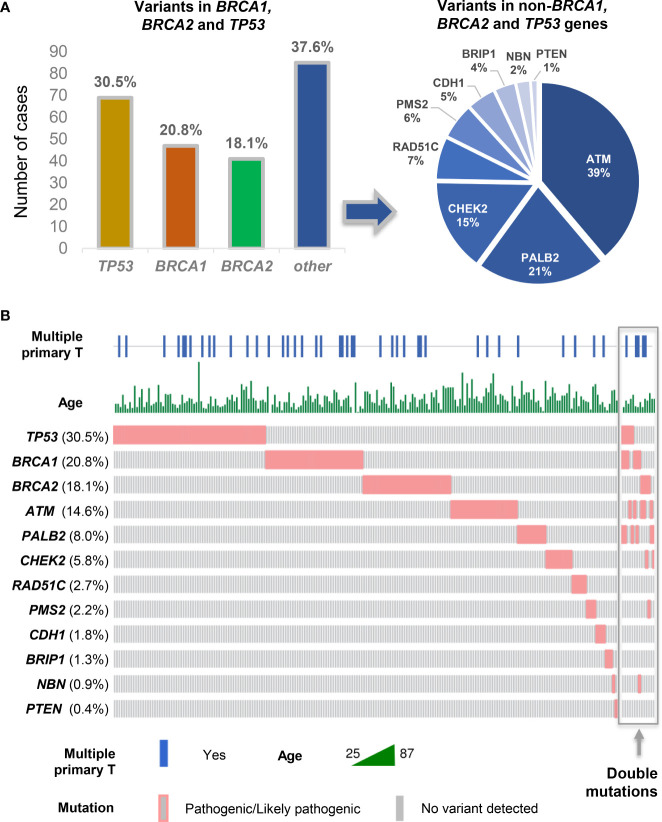
Germline variants detected in BC-risk genes in the 226 patients with BC from the Brazilian Hereditary Cancer Registry. **(A)** Proportion of germline P and LP variants in *TP53*, *BRCA1*, and *BRCA2* (35%) was the most frequent, followed by *ATM*, *PALB2*, and *CHEK2*. **(B)** Frequency and distribution of pathogenic germline variants identified in BC genes, and associations with age at BC diagnosis and occurrence of multiple primary tumors (diagram implemented with Oncoprinter, available at: https://www.cbioportal.org/oncoprinter).

In our cohort, carriers who presented a *BRCA1* variant, regardless of what was the other pathogenic variant gene, developed BC at earlier ages (44.7% ≤ 40 years of age; p = 0.022) and were associated with a higher risk for multiple primary tumors when compared with carriers of other gene variants (**Figure 2B**). *TP53* variant carriers presented earlier onset BC (43.9% ≤ 40 years of age; p = 0.043), regardless of what was the other pathogenic variant gene. *ATM c*arriers were associated with BC after age 40 (≤ 40 years in 51.8%; p = 0.025) (**Table 1**).

**Table 1:** Association between germline variants with age of BC diagnosis and occurrence of additional primary tumors (among 226 cases with positive variants in BC-risk genes).

Variants	Breast Cancer Onset			Multiple Primary Tumors		
	≤40 years	>40 years	P^#^	No	Yes	P^#^
*TP53*						
Negative	69 (43.9%)	88 (56.1%)	0.043	126 (80.3%)	31 (19.7%)	1.000
Positive	41 (59.4%)	28 (40.6%)		55 (79.7%)	14 (20.3%)	
*BRCA1*						
Negative	80 (44.7%)	99 (55.3%)	0.022	149 (83.2%)	30 (16.8%)	0.025
Positive	30 (63.8%)	17 (36.2%)		32 (68.1%)	15 (31.9%)	
*BRCA2*						
Negative	93 (50.3%)	92 (49.7%)	0.388	149 (80.5%)	36 (19.5%)	0.672
Positive	17 (41.5%)	24 (58.5%)		32 (78%)	9 (22%)	
*ATM*						
Negative	100 (51.8%)	93 (48.2%)	0.025	152 (78.8%)	41 (21.2%)	0.344
Positive	10 (30.3%)	23 (69.7%)		29 (87.9%)	4 (12.1%)	
*PALB2*						
Negative	103 (49.5%)	105 (50.5%)	0.465	165 (79.3%)	43 (20.7%)	0.538
Positive	7 (38.9%)	11 (61.1%)		16 (88.9%)	2 (11.1%)	
*CHEK2*						
Negative	106 (49.8%)	107 (50.2%)	0.255	170 (79.8%)	43 (20.2%)	1.000
Positive	4 (30.8%)	9 (69.2%)		11 (84.6%)	2 (15.4%)	
Other*						
Negative	99 (48.3%)	106 (51.7%)	0.820	164 (80%)	41 (20%)	1.000
Positive	11 (52.4%)	10 (47.6%)		17 (81%)	4 (19%)	
≥2 variants						
No	102 (48.1%)	110 (51.9%)	0.588	172 (81.1%)	40 (18.9%)	0.161
Yes	8 (57.1%)	6 (42.9%)		9 (64.3%)	5 (35.7%)	

*Variants in BRIP1, CDH1, NBN, PMS2, PTEN, and RAD51C; ^#^Fisher exact test.

### Prevalence of DH variant carriers

**Figure 3:**
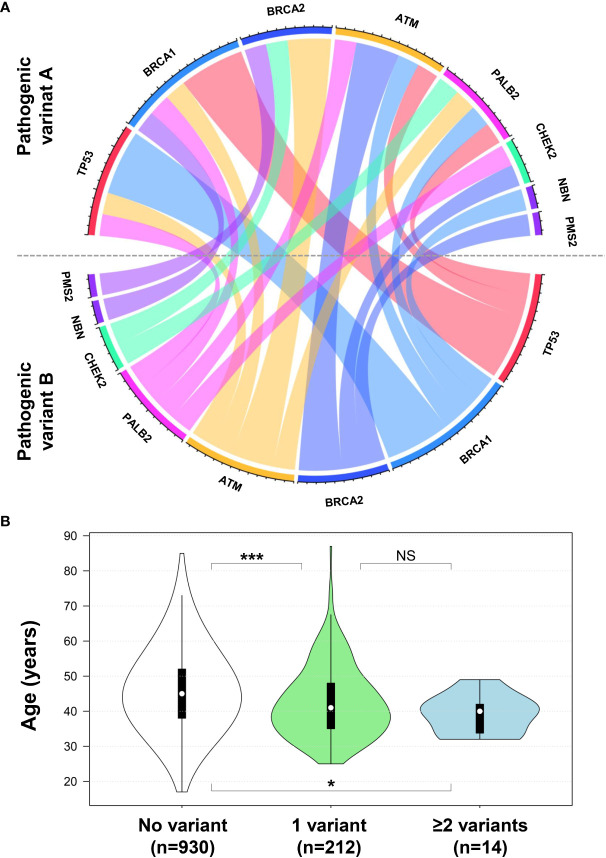
**(A)** Chord diagram illustrating the BC-risk genes combinations among germline DH pathogenic variants. **(B)** Violin plot diagram showing the distribution, interquartile range, and median age of BC diagnosis from cases with no variant (white), one variant (green), and two or more variants (blue) detected. ***P < 0.001; *P < 0.05; NS, not significant (Dunn’s *post hoc* test). Chord diagram and violin plot were implemented with the circlize and plotly R packages, respectively.

Individuals who presented with more than one germline variant had most germline pathogenic variants in *BRCA1* (6 of 14; 42.9%), *TP53* (5 of 14; 35.7%), and *BRCA2* (4 of 14; 28.6%) (**Figure 2A**). The co-occurrence of DH variants in high- or moderate-penetrance genes was not a common event, which was present in 1.2% (14 of 1,156) of all women with BC who had undergone gene panels (**Figure 2B**). Among carriers of at least one germline pathogenic/LP variant in a high-penetrance gene (*BRCA1* = 6; *PALB2* = 6; *TP53* = 5; *BRCA2* = 4), DH were found in 6.2% (14 of 226). Moderate-penetrance gene variants were detected in combination with the high-penetrance genes (*ATM* = 5; *CHEK2* = 2; *NBN =* 1; *PMS2* = 1) that were detected as DH with one high-penetrance gene variant (**Figure 3A**).

### Association between DH pathogenic variants, BC age of onset, and risk of multiple tumors

We sought to investigate whether the 14 carriers of more than one pathogenic/LP variant in BC-risk genes presented earlier BC and if there was a higher prevalence of multiple primary tumors. Difference of age of BC onset in patients with one germline pathogenic variant was not significant if compared with those with DH (48.1% vs. 57.1%, respectively, P = 0.588) (**Table 1**). The median age of BC diagnosis of patients with no germline variants and with one variant and two or more variants detected was 45, 41, and 40 years, respectively (**Figure 3B**). There was a higher risk for multiple primary tumors in carriers of DH variants compared with carriers of one variant but not statistically significant (35.7% vs. 18.9%, respectively, P = 0.161) (**Table 1**).

From the 14 individuals carrying more than one variant in BC-risk genes, we were able to identify the occurrence of a bilateral BC in 28.6% of all cases (4 of 14). All bilateral BC cases occurred in individuals who harbored either one *BRCA1* or a *BRCA2* variant. A second germline variant was found in *PALB2*, *CHEK2*, *ATM*, and *NBN*, and they presented an additional cancer at the ages of 37, 57, 52, and 58, respectively. In addition, patient 4 (*BRCA1/TP53* variants) presented a pancreatic adenocarcinoma at 40 years and lung cancer at 53 years. It is important to notice that two *BRCA1/PALB2* cases (Patients 1 and 2) also had a germline variant in the moderate-penetrance Brazilian founder variant in *TP53 gene* c.1010G>A (p.Arg337His) that is also found in patients 4, 11, and 12. From 10 individuals with available molecular BC subtypes information, seven of them were classified as Luminal, two as HER2+, and one as triple negative subtype (**Table 2**).

**Table 2:** Description of cases positive for DH mutation in high- and moderate-penetrance genes in BC.

Patient ID	Family ID	Detected Mutations	Age at BC	Molecular Subtype	Other Primary Cancers (Age)
1	F02114	*BRCA1 (c.5266dupC)*, *PALB2 (c.3271C>T)* and *TP53 (c.1010G>A)*	32	NA	No
2	F02114	*BRCA1 (c.5266dupC)*, *PALB2 (c.3271C>T)* and *TP53 (c.1010G>A)*	40	NA	No
3	F00620	*BRCA1 (c.4165_4166delAG)* and *PALB2 (c.1240C>T)*	32	NA	BC (37)
4	F00006	*BRCA1 (c.5266dupC)* and *TP53 (c.1010G>A)*	36	HER2+	Pancreas (40), Lung (53)
5	F00952	*BRCA1 (c.5266dupC)* and *ATM (c.6729_6730delAA)*	33	TNBC	No
6*	F01844	*BRCA1 (c.1687C>T)* and *NBN (c.1142delC)*	38	Luminal	BC (58)
7	FY0240	*BRCA2 (c.6024dup)* and *CHEK2 (c.478A>G)*	40	NA	BC (57)
8	F02417	*BRCA2 (c.2516dupA)* and *PMS2 (c.2182_2184delinsG)*	49	Luminal	No
9	FY0073	*BRCA2 (c.8960del)* and *ATM (c.901+1G>A)*	32	Luminal	No
10	FY0073	*BRCA2 (c.8960del)* and *ATM (c.901+1G>A)*	42	HER2+	BC (52)
11	FY0751	*TP53 (c.1010G>A)* and *PALB2 (c.2711G>A)*	47	Luminal	No
12	F01266	*TP53 (c.1010G>A)* and *ATM (c.4906C>T)*	42	Luminal	No
13	F01776	*PALB2 (c.2711 G>A)* and *CHEK2 (c.319+2T>A -Splice donor)*	42	Luminal	No
14	F02082	*PALB2 (c.3350+5G>A)* and *ATM (c.2140del)*	42	Luminal	No

BC, breast cancer; NA, not available; *also detected mutation in the MUTYH gene (c.536A>G).

### Triple pathogenic variant carriers in BC-risk genes

**Figure 4:**
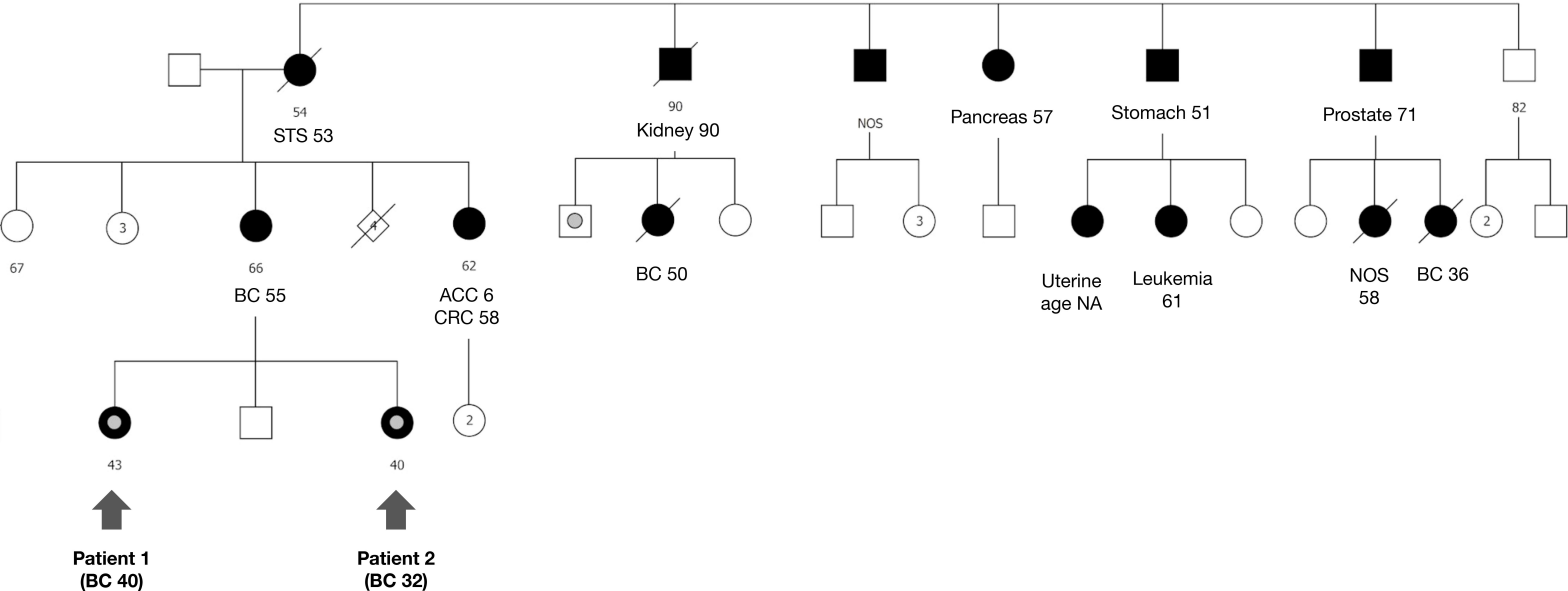
Triple pathogenic variant carriers in BC-risk genes. In family 02114, two individuals with early onset breast cancers are both carriers of three pathogenic variants in three cancer predisposing genes, BRCA1 c.5266dup (p.Gln1756fs), TP53 c.1010G>A (p.Arg337His) and PALB2 c.3271C>T (p.Gln1091Ter).

Among the DH cases, in one family, two individuals (sisters) carried three germline pathogenic variants in BC-risk genes (F02114), comprising *BRCA1 c.5266dup (p.Gln1756fs)*, *TP53 c.1010G>A (p.Arg337His)*, and *PALB2 c.3271C>T (p.Gln1091Ter).* Both sisters were affected by BC at a young age (32 and 40 years). Their mother, who was not tested, had a BC diagnosis at age 55. There is a wide tumor spectrum in the maternal side, including breast, adrenal, colon, kidney, leukemia, pancreas, prostate, sarcoma, skin, stomach, and uterus. No family history of cancer is reported in the paternal side (**Figure 4**).

## Discussion

As a result of the expansion toward larger gene panels and more individuals being tested, the identification of DH events is becoming more frequent. Currently, there are only a few reports in medical literature addressing the detection of DH (14, 18–22). It is still unknown whether the presence of a DH in high- and moderate-penetrance genes would have an additive or synergic effect on lifetime risk of tumor development. In this study, we conducted a large cancer predisposition gene panel in a cohost of early onset or familial BC. A total of 19.6% (226 of 1,156) of women with BC carried at least one pathogenic variant in a moderate- or high-penetrance BC-risk genes. DH variants in BC-moderate to high-risk genes were observed in a subset of 14 patients, representing 1.2% (14 of 1,156) of the BC cohort. Most of the DH carriers carried at least one pathogenic variant in *BRCA1* or *BRCA2* genes (10 of 14, 71.4%). A systematic review recently reported 385 individuals carrying pathogenic variants in two or more of 94 cancer susceptibility genes (14). Although more cancer-risk genes were considered and their cohort was not limited to BC, 74.5% (287 of 385) were also carriers of variants in *BRCA1* or *BRCA2* genes.

Pathogenic variants in *BRCA1*, *BRCA2*, and *TP53* w*e*re the most commonly found in this cohort, corresponding to 69.4% of all pathogenic variants (PV). PVs in *BRCA1* were detected in 20.8% (47 of 226) of all carriers, *BRCA2* in 18.1% (41 of 226), and *TP53* in 30.5% (69 of 226). A population-based study conducted in 32,247 women with BC screened for a panel of cancer predisposing genes found the majority of variants to occur in BRCA1 and BRCA2 genes. Nevertheless, limited numbers of women with pathogenic variants in *TP53* did not allow associations with BC in this large international cohort (PMID: 33471974). The high frequency of patients with BC carrying *TP53* variants in our cohort is probably due to the Brazilian founder variant c.1010G>A (p.Arg337His) present in 0.2%–0.3% of the South and Southeastern Brazilian population (6, 7). In fact, c.1010G>A (p.Arg337His) comprised 79.7% (55 of 69) of all *TP53* variants detected in our cohort. This study was conducted is the main reference center for Li-Fraumeni syndrome in the country, and this may have caused a recruitment bias.

In this cohort, carriers of *BRCA1* and *TP53* variants had BC at an early age of onset, whereas *ATM* variant carriers were more likely to be diagnosed later in life, in agreement with prior reports (23–25). Patients with BC who carry a germline pathogenic variant in *BRCA1* gene mutation are more likely to have triple-negative BC (TNBC) (PMID: 19298662). Previous studies have suggested that BC in TP53 pathogenic variant carriers is highly likely to be HER2 positive (PMID: 20805372).

The age of BC diagnosis in the DH carriers was similar to those with a high- or moderate-penetrance pathogenic variant in one gene. The BC onset in DH was comparable to reports of hereditary BC related to *BRCA1* and *TP53* germline variant carriers (26). Bilateral BC was found mostly in carriers of at least one *BRCA1* and/or *BRCA2* pathogenic variant (28%). In previous reports in the literature, the occurrence of bilateral BC in *BRCA1* and *BRCA2* carriers may be as high as 53% (27). Although *TP53* germline mutation carriers are at a higher risk of developing bilateral BC (28), carriers of DH with at least one *TP53* variant in this study did not present bilateral BC. Although there was a higher prevalence of bilateral BC in DH compared with carriers of a single pathogenic variant (35.7% vs. 18.9%, respectively), there was no putative additive effect of DH (p = 0.161).

The advances in DNA sequencing technologies and the decreased costs of genetic assays lead to expressive increase in the number of genes incorporated in genetic testing for BC risk prediction (20). Most gene panels include high-penetrance genes, such as *BRCA1*, *BRCA2*, *CDH1*, *PALB2*, *PTEN*, *STK11*, and *TP53*, and moderate-penetrance genes, such as *ATM*, *BRIP1*, *CHEK2*, *FANCD2*, *RAD51C*, *NBN*, and *PMS2*, which also associates with lifetime BC risk (29). Although the clinical impact of *NBN* and *PSM2* on BC susceptibility has remained uncertain, their introduction in clinical genetic testing for suspected hereditary BC has been recently supported (13, 30). *NBN* was shown to increase about three-fold the risk in BC (odds ratio, 3.1; 95% CI, 1.4–6.6) and indicates that the 657del5 deletion [c.657_661del (p.Lys219fs)] and perhaps the R215W substitution contribute to inherited BC susceptibility (11). We identified two cases harboring NBN pathogenic variants: the aforementioned c.657_661del (p.Lys219fs) and c.1142del (p.Pro381fs). Pathogenic variants in *PMS2* and BC risk remain a controversial association. In a large cohort of women with Lynch syndrome identified through panel testing, there was no evidence for increased risk of BC compared with the general US population (12). However, when evaluating by gene, the age-standardized BC risks for PMS2 (standard incidence ratio = 2.92; 95% CI, 2.17–3.92) were associated with a statistically significant risk for BC (30). In our cohort, we described one patient who carried a *BRCA2* (c.2516dupA) and *PMS2* (c.2182_2184delinsG) and who developed a BC at age 49.

In a cohort of 8,162 breast/ovarian cancer families from the German Consortium for Hereditary Breast and Ovarian Cancer, eight female DH patients for *BRCA1* and *BRCA2* mutations were described and analyzed for their phenotypic features (22). The authors did not find association of DHs with younger age at BC diagnosis compared with single heterozygous index patients. Nevertheless, it is suggested DH patients had a more severe disease than their female relatives carrying a single *BRCA1/2* mutation. Likewise, a study including 5,391 Slavic women carriers of *BRCA1*, *CHEK2*, *NBN*, *ATM*, and *BLM* variants identified 17 patients with DH BC. No differences in age of onset and risk of bilateral BC were found between single variant and DH carriers (18).

We identified three carriers of *ATM* who also had either a *BRCA1* or *BRCA2* variant. A *BRCA1/ATM* carrier (case 5) developed BC at a young age (33 years old) and *BRCA2/ATM* carriers (cases 9 and 10, FY0073) developed BC at 32 and 45 years, respectively. Case 10 developed bilateral BC (a second BC was diagnosed at 52 years). There are no reports in the literature indicating an additive effect of both germline variants in female patients. An *in vitro* study was performed to evaluate the combinations of wild type and hemizygous genotypes for *BRCA1* and *ATM* and its interference in cell transformation and apoptosis induced by radiation (31). The results indicated that double heterozygosity for *ATM* and *BRCA1* leads to an additive effect on tumor development than single heterozygosity. In a murine model of induced *BRCA1* and *ATM* DH loss, a synergistic effect in mammary gland tumorigenesis and tumor aggressiveness was observed. The results suggested that these DH combinations lead to a newly characterized developmental defect during glandular maturation (32). As only a few individuals in this study were found to carry DH in *BRCA1/ATM* or *BRCA2/ATM*, the same synergistic effect could not be confirmed.

In our cohort, three individuals who carried *BRCA1* and *PALB2* pathogenic variants (cases 1, 2, and 3) developed BC at young age (32, 40, and 32, respectively). Case 3 developed a second BC at 37 years. Multiple combinations of *BRCA1/2*, *PALB2*, and *TP53* variants were also tested in a murine mammary gland model using parallel conditional knockout (33). Curiously, the combined loss of *BRCA1* and *PALB2* was demonstrated to increase reactive oxygen species and apoptosis, which could be responsible for a delayed tumorigenesis in this condition. Carriers of multiple combinations of *BRCA1*, *PALB2*, and *TP53* did not present any differences than what is expected of carriers of only one pathogenic variant in any of the three genes. Although *in vitro* and *in vivo* models are useful to predict clinical outcomes, cases and series reports in humans have shown inconclusive results (34–36).

Curiously, we detected two patients with BC in our registry who carried three pathogenic variants in high-penetrance BC-risk genes. The presence of three pathogenic germline variants in the same individual is an extremely rare event, and there are only a few case reports in the literature (19, 21). In this cohort, two sisters (identified as patients 1 and 2) tested positive for concomitant *BRCA1 (c.5266dupC)*, *PALB2 (c.3771C>T)*, and *TP53 (c.1010G>A)* variants. They both had BC at early ages (≤40 years). Further molecular studies on family members were not performed, although multiple cancers of the Li-Fraumeni and *BRCA1/BRCA2*-spectrum were referred in the maternal family history.

However, we believe that DH carriers may benefit from more intensive surveillance programs/follow-up care and family members should perform cascade testing. Risk-reducing procedures must be well integrated and individualized in these populations.

In conclusion, the multi-gene hereditary cancer panel testing comes with the challenge of counseling and managing patients who may carry variants in multiple germline cancer susceptibility genes. Functional studies should be conducted to improve our comprehension of how germline variant combinations may interact and impact in the tumor development, reflecting on the BC onset and increased risk for multiple tumors. Larger studies are needed to estimate whether the presence of DH has a synergistic interaction or additive effect on cancer risk in BC. It is important to investigate these rare DH genotypes to provide better personalized clinical management for carriers and determine the benefit of risk-reducing procedures in these populations.

## Data availability statement

The raw data supporting the conclusions of this article will be made available by the authors, without undue reservation.

## Ethics statement

This study was reviewed and approved by Research Ethics Committee (ID1747) and the National Research Ethics Committee (number 39512620.9.0000.5461). The patients/participants provided their written informed consent to participate in this study.

## Author contributions

Concept and design: TM, MB-F, and MA. Acquisition, analysis, or interpretation of data: TM, MB-F, JP, and MA. Drafting of the manuscript: TM, MB-F, and MA. Critical revision of the manuscript for important intellectual content: TM, MB-F, JP, and MA. Supervision: MA. All authors have read, edited and approved the final manuscript, and agree to be accountable for the content of the work.

## Funding

This work was supported by Stand Up to Cancer (SU2C - subaward number 0266-3190-4609).

## Conflict of interest

The authors declare that the research was conducted in the absence of any commercial or financial relationships that could be construed as a potential conflict of interest.

## Publisher’s note

All claims expressed in this article are solely those of the authors and do not necessarily represent those of their affiliated organizations, or those of the publisher, the editors and the reviewers. Any product that may be evaluated in this article, or claim that may be made by its manufacturer, is not guaranteed or endorsed by the publisher.
